# Integrated Blood Biomarker and Neurobehavioural Signatures of Latent Neuroinjury in Experienced Military Breachers Exposed to Repetitive Low-Intensity Blast

**DOI:** 10.3390/ijms27020592

**Published:** 2026-01-06

**Authors:** Alex P. Di Battista, Maria Y. Shiu, Oshin Vartanian, Catherine Tenn, Ann Nakashima, Janani Vallikanthan, Timothy Lam, Shawn G. Rhind

**Affiliations:** 1 Toronto Research Centre, Defence Research and Development Canada, Toronto, ON M3K 2C9, Canada or dibattista.alex@gmail.com (A.P.D.B.); maria.shiu@ecn.forces.gc.ca (M.Y.S.); or oshinv1@mac.com (O.V.); janani.vallikanthan@ecn.forces.gc.ca (J.V.); timothy.lam@ecn.forces.gc.ca (T.L.); 2 Faculty of Kinesiology and Physical Education, University of Toronto, Toronto, ON M5S 2W6, Canada; 3 Department of Psychology, University of Toronto, Toronto, ON M5S 2W6, Canada; 4 Suffield Research Centre, Defence Research and Development Canada, Medicine Hat, AB T1A 8K6, Canada; catherine.tenn@ecn.forces.gc.ca

**Keywords:** axonal injury, blast overpressure, brain health monitoring, traumatic brain injury, military breachers, occupational exposure, subconcussive neurotrauma, blood biomarkers, GFAP, neurofilament light chain, tau protein, Bayesian latent-variable modeling, working memory, Rivermead Post-Concussion Symptoms Questionnaire

## Abstract

Repeated exposure to low-level blast overpressure (BOP) during controlled detonations is an emerging occupational health concern for military breachers and Special Operations Forces personnel, given accumulating evidence that chronic exposure may produce subtle, subclinical neurotrauma. This study derived a latent neuroinjury construct integrating three complementary domains of brain health—post-concussive symptoms, working-memory performance, and circulating biomarkers—to determine whether breachers exhibit coherent patterns of neurobiological alteration. Symptom severity was assessed using the Rivermead Post-Concussion Questionnaire (RPQ), and working memory was assessed with the N-Back task and a panel of thirteen neuroproteomic biomarkers was measured reflecting astroglial activation, neuronal and axonal injury, oxidative stress, inflammatory signaling, and neurotrophic regulation. Experienced Canadian Armed Forces breachers with extensive occupational BOP exposure were compared with unexposed controls. Bayesian latent-variable modeling provided probabilistic evidence for a chronic, subclinical neurobiological signal, with the strongest contributions arising from self-reported symptoms and smaller but consistent contributions from the biomarker domain. Working-memory performance did not load substantively on the latent factor. Several RPQ items and circulating biomarkers showed robust loadings, and the latent neuroinjury factor was elevated in breachers relative to controls (97% posterior probability). The pattern is broadly consistent with subclinical neurobiological stress in the absence of measurable cognitive impairment, suggesting early or compensated physiological alterations rather than overt dysfunction. This multidomain, biomarker-informed framework provides a mechanistically grounded and scalable approach for identifying subtle neurobiological strain in military personnel routinely exposed to repetitive low-level blast. It may offer value for risk stratification, operational health surveillance, and the longitudinal monitoring of neurobiological change in high-risk occupations.

## 1. Introduction

Explosive breaching, a core tactic for gaining rapid entry and tactical advantage, exposes military personnel to repeated low-level blast overpressure (BOP) generated by user-directed munitions during controlled detonations [1,2]. Within the Canadian Armed Forces (CAF), tactical breachers represent one of the most chronically exposed groups, often performing multiple breaches per day across successive training cycles and operational deployments [3]. Although single events typically fall below diagnostic thresholds for traumatic brain injury (TBI) or concussion, emerging evidence indicates that repeated subconcussive blasts can produce subtle, cumulative alterations in central nervous system (CNS) structure and function [4,5,6,7,8,9,10]. Over time, this additive exposure burden has been linked to measurable disturbances in cognition, balance, sleep regulation, and hearing [11,12,13,14,15,16], as well as elevated rates of depression, post-traumatic stress disorder (PTSD), and other neurobehavioral symptoms [17,18,19,20,21].

BOP exposure begins with the rapid transmission of a shock front generated by explosive detonation, producing an instantaneous pressure rise followed by a nonlinear decay [22,23]. Propagating at supersonic speed, this primary blast wave imparts complex baromechanical loads to the body and skull, driving shear, tensile, and rotational forces through cerebrospinal fluid (CSF) and cranial tissues that induce diffuse axonal injury (DAI), blood–brain barrier (BBB) disruption, and microvascular strain [24,25,26,27,28]. Once delivered to the head, blast-induced loading may involve direct transmission, skull flexure, rapid head acceleration, and CSF cavitation—mechanisms consistently implicated in both human and experimental models of blast neurotrauma [29,30]. Although this mechanism remains controversial, some evidence suggests a potential thoracic-mediated contribution to blast neurotrauma, whereby pressure-wave transmission through the systemic circulation may indirectly stress cerebral and perivascular structures, producing transient cerebrovascular pressure surges that elevate intracranial pressure, compromise BBB integrity, and impair glymphatic clearance of neurotoxic proteins such as amyloid (A)-β and tau [31,32,33,34,35,36,37,38]. These primary mechanical insults are thought to initiate secondary cascades involving neuroinflammation, oxidative stress, astroglial activation, endothelial dysfunction, and disrupted neurovascular coupling [33,39,40,41,42,43,44,45], ultimately destabilizing synaptic signaling and metabolic homeostasis [46,47,48,49]. With repeated exposures, these perturbations accumulate into diffuse network dysfunction rather than focal lesions, manifesting clinically as cognitive fatigue, slowed processing speed, irritability, and affective dysregulation commonly reported among experienced breachers and other high-exposure personnel [13,18,19,50,51,52].

Blast-related neurotrauma is frequently accompanied by the release of brain-enriched proteins into the peripheral circulation, reflecting mechanical stress, astroglial activation, axonal strain, and disruption of neurovascular homeostasis [11,53,54,55,56,57,58]. Quantifiable using ultrasensitive immunoassays, these circulating biomarkers provide a minimally invasive means of detecting subtle neuronal, glial, and vascular perturbations even when conventional structural neuroimaging appears normal [5,53,54,55,56]. Collectively, they index complementary injury domains, including neuronal soma and dendritic injury (UCH-L1, NSE, NRGN, VILIP-1), axonal degeneration (NfL, pNF-H), astroglial activation and BBB disruption (GFAP, S100β), oxidative and mitochondrial stress (PRDX-6, CK-BB), inflammatory signaling (MCP-1), and neurotrophic regulation (BDNF) [57,58,59]. Within this expanding biomarker landscape, A-β40/42 peptides and phosphorylated (p)-tau isoforms have emerged as particularly sensitive indicators of cumulative CNS stress and early degenerative change, offering a plausible mechanistic link between repetitive subconcussive blast exposure and longer-term neurodegenerative risk [60,61,62,63,64]. Together, these neurobiological measures support a mechanistically grounded and scalable framework for probabilistic risk stratification and longitudinal monitoring of blast-related neurobiological strain, with potential relevance for evidence-informed return-to-duty decisions and individualized exposure management in high-risk military populations [65,66,67].

Despite major advances, most studies remain constrained by narrow molecular biomarker panels and a focus on acute, single-event exposures [67,68,69,70]. The chronic evolution of biomarker expression under career-long, low-level exposure remains poorly characterized [17,53]. Conventional imaging and behavioral tools lack the sensitivity to capture diffuse microscopic or network-level dysfunction [5,71]. In this context, circulating biomarkers offer a biologically grounded complement to neuroimaging and neurocognitive assessment, potentially capturing subclinical injury processes [69,72,73,74]. However, systematic investigations linking biomarkers with cognitive and symptomatic domains remain limited, particularly in studies of long-term exposure among high-risk occupational groups.

In this study, we extend prior research in CAF breachers and range staff showing persistent post-concussive symptoms, slowed cognitive–motor performance, and poorer self-reported health compared with unexposed controls [12,14,74]. These findings suggest that low-level BOP exposure may produce a latent, cumulative form of neurological stress spanning behavioral, cognitive, and molecular domains [45,75,76]. To further characterize these effects, we integrated three complementary dimensions of brain health—self-reported symptoms, working-memory performance, and biomarkers of astroglial activation, axonal injury, oxidative stress, inflammation, and neurotrophic regulation. Using a Bayesian latent-variable framework, we tested whether breachers with chronic BOP exposure exhibited higher latent neuroinjury scores than unexposed controls and identified the symptom and neurobiological features contributing most to this construct [77]. This probabilistic approach aims to estimate subclinical CNS perturbations that cannot be directly measured by capturing shared variance across behavioral, cognitive, and biomarker domains—offering a mechanistically integrated alternative to conventional analyses [78]. Within this model, biomarkers index neuronal and glial stress, symptoms reflect functional impact, and N-Back performance represents frontoparietal network efficiency; regions vulnerable to blast-related microstructural disruption [79,80]. This multidomain Bayesian framework improves our understanding of blast-related neurobiological change, highlights potential indicators of subclinical injury, and establishes a scalable foundation for precision monitoring of occupational brain health in military and other high-risk groups repetitively exposed to low-level blast.

## 2. Results

### 2.1. Participant Characteristics

Participant demographics and service characteristics are summarized in Table 1. Both blast-exposed breachers and unexposed controls were predominantly male (89% each), with a median age of 32 years (IQR: 26–37). Breachers were primarily senior-ranking personnel (61%), evenly split between Regular Force and Primary Reserve, and reported a median of 6.5 years (IQR: 4–10) of breaching experience. Nearly two-thirds (63%) had prior war-zone deployments. In contrast, controls were more frequently Primary Reserve members (58%), mostly junior NCMs (68%), and had no prior deployments.

N-Back working-memory scores were comparable between groups across all task levels. Group-level summaries for biomarker distributions (Low/Medium/High) and RPQ symptom severity categories (Low/Moderate/Severe), as well as associated cut-points, are provided in Supplementary Tables S1 and S2.

### 2.2. Item Contributions to the Latent Neuroinjury Score

**Figure 1:**
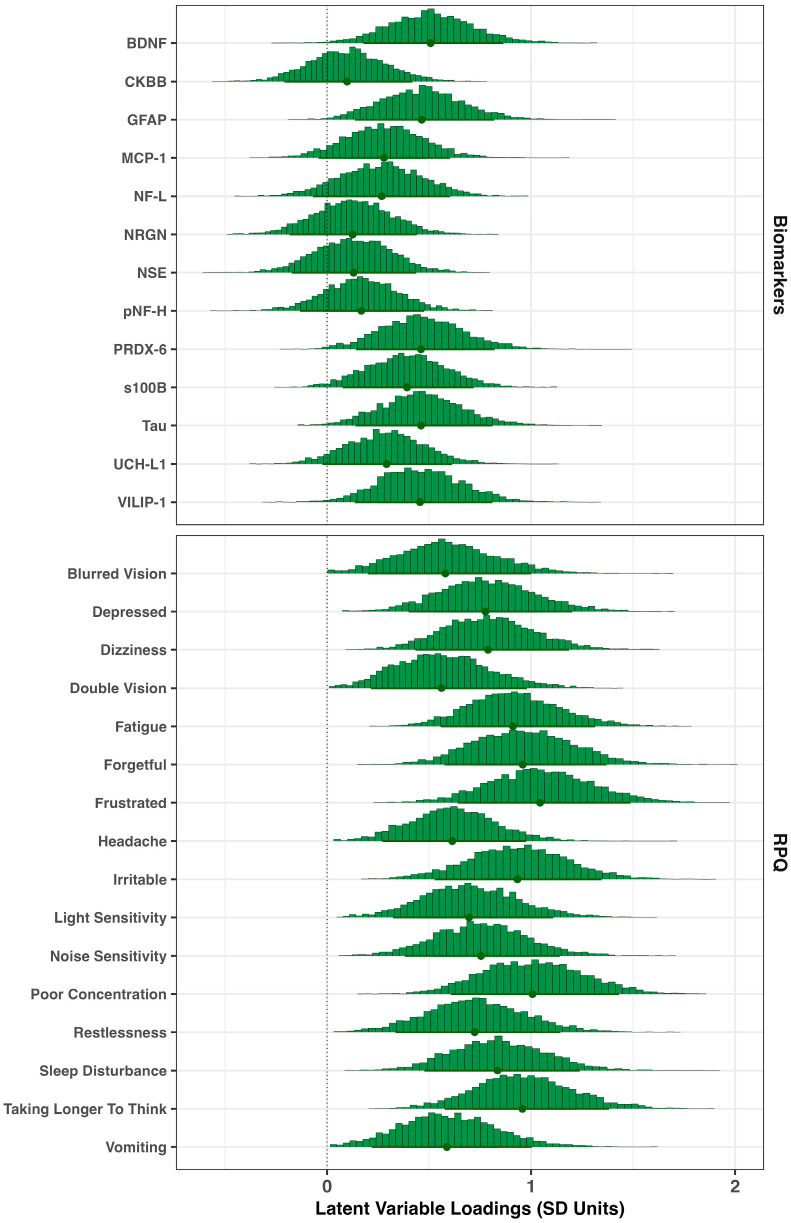
Item loadings on latent neuroinjury. Posterior histograms for item loadings linking biomarkers and RPQ symptoms to the latent neuroinjury factor. Higher magnitudes indicate stronger associations; positive values denote greater symptom severity (or poorer task performance) or biomarker concentration with higher latent neuroinjury. Dots represent the posterior means for each loading, and the thicker line across the bottom represents the 90% credible interval.

The posterior histograms of the per-item loadings on the latent neuroinjury variable are shown in Figure 1. Among the three domains, symptom measures from the RPQ were the strongest contributors to the latent neuroinjury score (mean loading = 0.8 SD units; 90% Credible Interval [CrI]: 0.3–1.3). Biomarker variables contributed moderately (mean = 0.3; 90% CrI: –0.1–0.7), while working-memory performance (not shown on the plot) contributed minimally (mean = 0.1; 90% CrI: 0–0.2). Within the RPQ domain, ‘Frustration’, ‘Forgetful’, ‘Poor Concentration’, and ‘Taking Longer to Think’ showed the strongest associations, while top biomarker contributors included BDNF, GFAP, PRDX-6, Tau, and VILIP-1.

### 2.3. Latent Neurological Injury Scores in Breachers and Controls

**Figure 2:**
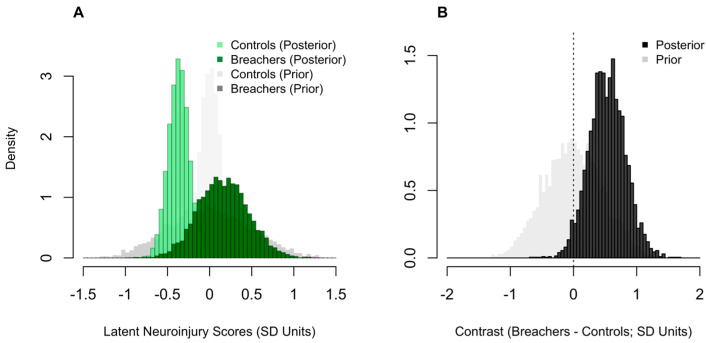
Comparison of latent neuroinjury scores between Breachers and Controls. Posterior histograms of latent neuroinjury scores (**A**); standard-deviation, SD units) for Controls and Breachers, with corresponding prior densities overlaid in lighter grey colouring. Posterior histogram of the group contrast (**B**); Breachers–Controls, SD units), with the prior contrast shown in light grey. The vertical line marks zero (no difference). Higher values indicate greater latent neuroinjury.

Posterior densities estimates for the group-level latent neuroinjury scores in Breachers and Controls can be seen in Figure 2. The posterior probability quantifies how likely it is, given the data and model assumptions, that one group’s score exceeds another. Breachers showed a higher latent neuroinjury score than controls, with a 97% posterior probability that the group difference was greater than zero (pprob > 0). The mean latent score for Breachers was 0.2 SD units (90% CrI: −0.3 to 0.7), compared with −0.4 SD units in controls (90% CrI: −0.6 to −0.2). Although the absolute difference was modest, the high posterior probability supports a consistent shift toward greater neurobiological stress in the blast-exposed cohort.

### 2.4. Practical Implications of Elevated Neuroinjury Scores

To illustrate how elevated latent neuroinjury manifests across domains, we generated model-based predictions for biomarker concentrations, memory performance, and RPQ symptom severity. These estimates quantify the expected expression of neuroinjury, captured by the latent model, across biological and symptomatic measures, contrasting Breachers and Controls. Unlike the raw group-level summaries presented in Table 1 and the supplementary biomarker (Table S1) and RPQ (Table S2) data, the model-based predictions isolate neuroinjury-specific variance by accounting for shared covariance across domain items. In doing so, they distinguish variance attributable specifically to the underlying latent process from that arising from uncontrolled sources of individual or measurement variability.

**Figure 3:**
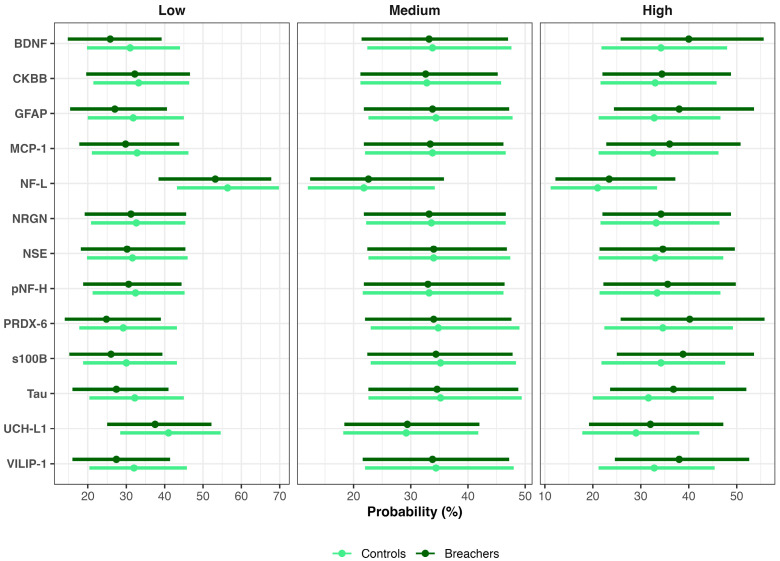
Posterior probabilities of biomarker concentration categories in Breachers and Controls. Bayesian posterior mean probabilities (points) and 90% credible intervals (bars) for low, medium, and high biomarker concentrations in Breachers (dark green) and Controls (light green).

For instance, consistent with higher latent neuroinjury scores in Breachers, the model predicted a greater estimated probability of ‘High’ BDNF concentrations in Breachers (>328.5 pg/mL): 40% (90% CrI = 26–56%) versus Controls (mean estimate: 35%, 90% CrI: 22–48%), representing an average difference of 6% (90%, 0–15%; pprob = 88%). Conversely, low BDNF concentrations (<181.3 pg/mL) were less likely in Breachers (26%, 15–39%) than in Controls (32%, 20–44%), a 5% difference (90% CrI: −2–13%; pprob = 89%). There was no meaningful difference between groups in the probability of medium BDNF concentrations (181.3–328.5 pg/mL). These results indicate that group differences in BDNF are stable but slight, and concentrated at the higher end of the concentration range, rather than reflecting a uniform shift across all levels. Similar distributional patterns were observed for GFAP, VILIP-1, Tau, and PRDX-6 (Figure 3).

**Figure 4:**
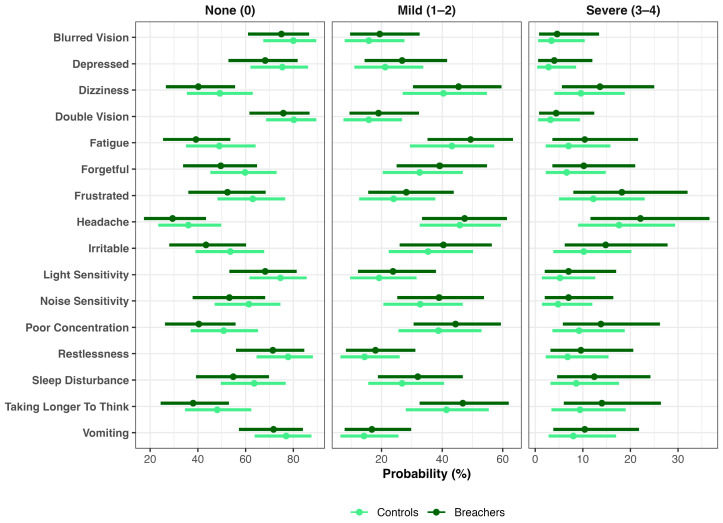
Distributional shifts in post-concussive symptoms by group. Bayesian ordinal models estimated the probability of endorsing None (0), Mild (1–2), or Severe (3–4) symptom levels for each Rivermead Post-Concussion Questionnaire (RPQ) item in Breachers and Controls. Points represent posterior means and horizontal bars denote 90% credible intervals.

Parallel analyses of RPQ items demonstrated that Breachers were more likely to endorse frustration across all severity levels, reflecting a rightward shift in symptom distribution. The probability of reporting no frustration (“None”) was lower in Breachers (52%, 36–68%) than in Controls (63%, 48–77%), a 10% difference (90% CrI: 0–22%; pprob = 95%). Mild frustration (scores 1–2) was slightly more frequent among Breachers (29%, 16–44%) compared with Controls (24%, 13–38%), a 4% difference (90% CrI: 2–11%; pprob = 88%), while severe frustration (scores 3–4) occurred in 19% (8–32%) of Breachers versus 13% (5–23%) of controls (Δ = 6%; 90% CrI: 0–14%; pprob = 93%). Collectively, these findings indicate that Breachers were less likely to be symptom-free and more likely to report either mild or severe frustration, consistent with higher latent neuroinjury scores. Similar rightward-shifted distributions were observed for other RPQ items, including Poor Concentration, Taking Longer to Think, Irritability, Noise Sensitivity, Forgetfulness, and Depression (Figure 4).

**Figure 5:**
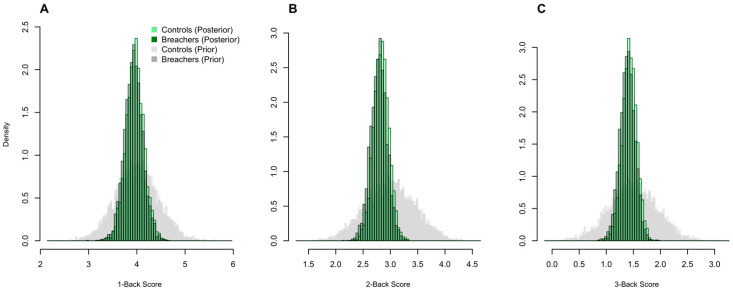
Working-memory performance does not differ between Breachers and Controls. Posterior histograms of N-Back sensitivity (*d′*) for 1-Back (**A**), 2-Back (**B**), and 3-Back (**C**) conditions show substantial overlap between Breachers and Controls, with no meaningful group differences in accuracy or sensitivity. Corresponding prior histograms overlaid in grey.

Despite group-level differences in symptom reporting and biomarker profiles, no differences were observed in N-Back working-memory performance between Breachers and Controls. This suggests that subjective symptom burden and underlying neurobiological alterations may emerge independently of overt cognitive impairment detectable by standard behavioral testing (Figure 5).

## 3. Discussion

### 3.1. Overview and Principal Findings

This study provides convergent probabilistic evidence that chronic occupational exposure to low-level BOP among experienced breachers is associated with a latent pattern of subclinical neurobiological stress. Using a Bayesian latent-variable framework, we integrated biomarker, symptom, and cognitive domains into a unified latent neuroinjury construct. Breachers exhibited higher latent scores than military controls with a high posterior probability (97%). This elevation was driven primarily by post-concussive symptom burden—most notably difficulties with concentration, irritability, forgetfulness, and slowed thinking—with more modest contributions from circulating biomarkers indexing astroglial activation (GFAP), oxidative stress (PRDX-6), synaptic and neurotrophic signaling (VILIP-1, BDNF), and tau-related cytoskeletal strain. In contrast, working-memory performance contributed minimally to the latent construct, consistent with preserved task-level accuracy despite underlying symptomatic and biological differences. Posterior predictive contrasts further revealed rightward shifts in symptom distributions and subtle, non-uniform biomarker alterations in breachers—patterns that may reflect cumulative astroglial–axonal stress and compensatory neural adaptation, while acknowledging that alternative explanations cannot be excluded. Although exploratory, this work represents one of the most comprehensive multimodal assessments of circulating neurobiomarkers in a highly exposed operational cohort to date and highlights the utility of probabilistic modeling for characterizing subtle neurobiological variation in complex military populations exposed to repetitive blast.

### 3.2. Alignment with Prior Human Blast Biomarker Studies

The present findings align with a growing body of human research showing that both acute and chronic BOP exposure elicit quantifiable perturbations in astroglial, axonal, vascular, and neurotrophic proteins [17,53,67]. Although effect sizes were modest and inter-individual variability was evident, the biomarker pattern observed in this breacher cohort is broadly consistent with established pathophysiological processes implicated in blast-related neurotrauma. Experimental and human studies suggest that primary blast waves impart shear, tensile, and compressive forces to neural and vascular tissues, altering endothelial tight junctions and compromising BBB stability, with downstream consequences for astroglial activation, oxidative balance, and peripheral biomarker release [28,81,82]. Controlled live-fire, breaching, and heavy-weapons investigations consistently demonstrate rapid post-blast elevations in canonical CNS-derived biomarkers—including GFAP, UCH-L1, total and phosphorylated tau, and NfL—within hours to days after detonation [56,65,68,83]. 

Early field investigations established the translational sensitivity of these markers. Tate et al. first reported acute elevations in GFAP, UCH-L1, and αII-spectrin breakdown products in New Zealand breachers, with increases correlating with slowed reaction time and transient symptoms [11]. Carr et al. replicated acute UCH-L1 elevations in U.S. Army breachers over a two-week course—evidence of reproducible neuronal–glial stress in the absence of diagnosed concussion [72]. In a 5-year follow-up of the New Zealand cohort, Kamimori et al. observed no chronic change in GFAP or UCH-L1 among breachers repeatedly exposed to <4 psi, suggesting a physiological threshold separating safe from cumulatively injurious exposure [84]. Using ultrasensitive immunoassays, Boutté et al. found acute increases in tau, NfL, and Aβ40/42 with reduced GFAP within one-hour post-blast, correlating with reaction-time slowing [85]. Edwards et al. similarly reported biphasic tau and NfL responses after moderate blast (>5 psi), consistent with transient cytoskeletal disruption followed by delayed repair [73].

Comparable kinetics appear across operational contexts. Thangavelu et al. observed increased Aβ42 and reduced GFAP over three days of 0.50-caliber sniper training, linking impulse exposure to amyloidogenic processing and altered glial responsivity [86]. Tschiffely et al. reported acute GFAP reductions in experienced breachers that scaled inversely with cumulative exposure, suggesting adaptive or exhausted astroglial release dynamics under repeated low-level BOP [87]. Eonta et al. documented acute post-blast rises in GFAP and UCH-L1 after moderate BOP [50]. The magnitude of GFAP (~1.6-fold) and UCH-L1 (~3-fold) elevations in our chronically exposed breachers parallels these acute effects, implying that transient molecular responses may consolidate into altered steady-state levels with repeated exposure.

Beyond classical injury markers, convergent inflammatory and vascular signatures have been repeatedly observed. Gill et al. showed that moderate blast (~8 psi) down-regulated amyloid precursor protein and transiently elevated IL-6 and TNF-α [88,89]. Wang et al. identified down-regulation of Aβ40/42 alongside up-regulation of vascular and immune genes (THBS1, MMP9, CLU, LRP1) in U.S. breachers [67]. These signatures parallel BBB stress, altered endothelial signaling, and impaired clearance pathways reported in experimental models, including increases in S100B, MBP, NF-H, oxidative vascular injury, and microglial priming [90,91]. Elevated PRDX-6 and MCP-1 in our cohort are consistent with these processes, supporting ongoing oxidative and endothelial stress under repeated low-level BOP.

With increasing cumulative exposure, transient perturbations may progress toward chronic dysregulation. Career breachers, artillery operators, and SOF personnel demonstrate sustained elevations in GFAP, NfL, tau, and S100β proportional to exposure duration and symptom burden [5,92,93]. Agoston et al. reported prolonged elevations of GFAP, UCH-L1, claudin-5, occludin, MMP-9, IL-6, and brain-reactive autoantibodies following heavy-weapons training, suggesting persistent glio-vascular remodeling [94]. Rhind et al. documented chronic increases in anti-GFAP autoantibodies and endothelial injury markers, reinforcing BBB compromise and neuroimmune activation [45,74]. Edwards et al. further demonstrated that neuronal extracellular vesicle–associated tau and NfL tracked neurobehavioral symptom burden [92]. Longitudinal ADVANCE-TBI data show GFAP elevations persisting up to eight years post-injury [95]. Consistent with these reports, our cohort displayed parallel astroglial, axonal, and oxidative signatures within a single operationally exposed population.

Larger and multimodal studies converge on tau-related risk. In 106 active-duty personnel, Boutté et al. reported elevations in tau, UCH-L1, and Aβ40/42 proportional to cumulative impulse exposure [69]. Dickstein et al. linked blast exposure to tauopathy and axonal injury in veterans, demonstrating increased [^18^F]flortaucipir binding, elevated NfL, and perivascular tau deposition with astroglial scarring [96]. Vorn et al. observed acute increases in p-tau181, T-tau, and NfL during live-fire training [97]. Gilmore et al. associated cumulative blast exposure with elevated plasma p-tau181, neuroinflammatory markers, and increased cortical tau binding [98]. The mean plasma tau concentration in our breachers approximates ranges reported acutely post-blast, suggesting maintenance of low-grade cytoskeletal stress with repeated exposure.

The PRDX-6 elevation observed here extends prior evidence of glial and vascular activation to include mitochondrial redox stress, consistent with human breacher data and experimental models [29,50]. Although human data on VILIP-1 and BDNF in blast-exposed military populations remain limited, animal models and collision-sport studies demonstrate parallel dysregulation of calcium signaling and neurotrophic pathways [29,99]. Di Battista and colleagues, together with Buonora et al., reported elevations in PRDX-6, BDNF, and MCP-1 following mild TBI and concussion, linking oxidative stress and altered neurotrophic signaling to neuroinflammatory activation and delayed recovery [58,100,101]. Similar vascular–oxidative–neurotrophic patterns appear in breacher and SOF cohorts with sustained elevations of GFAP, NfL, tau, and endothelial activation markers [89]. These networks parallel microglial activation, cortical thinning, and astroglial scarring reported in MRI, PET, and postmortem studies [5,96,102,103]. In our cohort, elevated BDNF may reflect compensatory neurotrophic adaptation, while increased VILIP-1 suggests calcium-dependent synaptic stress—patterns paralleling working-memory inefficiencies and supporting a shared oxidative–neurotrophic axis across blunt and blast neurotrauma [65,104,105].

Together, convergent molecular, imaging, and neuropathological evidence outlines a reproducible vascular–glial–axonal continuum under chronic low-level blast exposure. The integrated biomarker signature observed here, GFAP, UCH-L1, tau, NfL, S100β, PRDX-6, VILIP-1, and BDNF, captures biological processes repeatedly documented in acute paradigms but expressed in a more persistent form. Modeled within a Bayesian latent-variable framework, these biomarkers clustered with symptom probability distributions and exceeded single-exposure magnitudes, consistent with cumulative neurobiological burden. Collectively, these findings support a cautious model in which repetitive low-level blast exposure may contribute to sustained vascular, glial, and metabolic stress, while underscoring the need for longitudinal, multimodal confirmation before causal inferences can be drawn.

### 3.3. Methodological and Analytical Advances

We implemented a principled Bayesian workflow designed to model the narratively generative data process through which occupational BOP exposure may give rise to neuroinjury across multiple domains. A strength of this approach (outlined in the ‘Workflow’ section of the methods) is its transparency and replicability: all scientific and statistical assumptions were made explicit, allowing other researchers to scrutinize, extend, or challenge the model directly; all our inferential claims are fully conditional on the assumptions we explicitly specified.

Our framework builds on the philosophical foundation articulated by I.J Good and others [106], who argued that probability represents a rational degree of belief rather than an objective property of the world. Accordingly, we emphasize that scientific rigor arises not from claims of objectivity, but from making our subjective modeling assumptions explicit and reproducible. Methodologically, we followed a principled Bayesian workflow espoused most prominently by Betancourt [107] and McElreath [108], including prior simulations, prior predictive checks, fake-data simulations, and visual posterior predictive validation—all of which are publicly available in our GitHub repository (*see Data Availability Statement*).

We view this iterative workflow as essential to professionalizing statistical practice in research. While it does not preclude errors in scientific reasoning and statistical inference, it promotes transparency, facilitates replication and iterative improvement in future studies, and reduces the risk of drawing nonsensical or misleading conclusions that have historically impeded scientific progress.

### 3.4. Reconciling “Normal” Cognition with Subclinical Neural Stress

Despite the robust symptom and biomarker signals in the present cohort, breachers did not differ from controls on conventional indices of working-memory accuracy or speed. This dissociation between molecular pathology and apparently intact behavior underscores a central challenge in studying subconcussive blast exposure: most neuropsychological tools were designed to detect overt impairment, not the diffuse, small-effect, network-level inefficiencies that accrue under chronic low-level BOP [12,13,109,110,111,112,113]. Aggregate scores on tasks such as the N-back can remain within normative bounds even as distributed cortical and subcortical systems operate at increased metabolic cost and reduced efficiency [114,115]. “Normal” performance may therefore be sustained by compensatory recruitment of additional neural resources—masking emerging dysfunction until physiological reserves are exceeded [116,117].

Multiple lines of evidence support this compensation-without-decompensation model in blast-exposed populations [17,118,119]. Consistent with this view, Di Battista et al. [120] recently applied Bayesian factor-analytic modeling to a larger cohort of CAF breachers, snipers, and military controls and found that, although individual task scores on four-choice reaction time, delayed matching-to-sample, N-back, and Stroop largely fell within normative ranges, snipers exhibited lower latent neurocognitive status than both breachers and controls, with effects driven primarily by higher-load working-memory demands. Complementing this, Martindale et al. [21] reported in a large LIMBIC-CENC cohort that low-level blast exposure did not independently predict decrements on standard cognitive tests but interacted with PTSD and deployment-related mild TBI to selectively worsen memory and processing speed, indicating that subtle blast-related vulnerability may emerge most clearly when network load is compounded by comorbid stressors. Early operational studies using computerized screening batteries (e.g., ANAM) likewise reported minimal group-mean decrements despite elevated subjective complaints [121,122,123,124]. Subsequent neuroimaging shows that such behavioral “normality” can coexist with reduced white-matter integrity, cortical thinning, and altered network topology within executive and attention systems [5,102,113,125,126,127]. Task-based fMRI reveals fronto-parietal hyperactivation and broader spatial recruitment during working-memory paradigms—signatures of compensatory up-regulation to maintain output [13,128,129,130].

Kashou et al.’s systematic review similarly emphasizes that resting-state fMRI findings in TBI are mixed and often subtle, with connectivity alterations emerging most clearly when analyses or tasks stress large-scale networks, further highlighting why early dysfunction may not appear on standard cognitive indices. For example, Pagulayan et al. [111] showed that Veterans with blast-related mTBI display widespread resting-state hyperconnectivity in frontal working-memory networks despite normal behavioral performance—a pattern interpreted as compensatory recruitment to maintain working-memory function. Consistent with this view, recent imaging reveals persistent resting-state hyperconnectivity in chronically exposed personnel and transient hypoconnectivity after acute blast—a dynamic pattern interpreted as adaptive reconfiguration under load [131,132,133]. Supporting this, Lam et al. [130] demonstrated a similar biphasic network pattern: in a large CAF breacher–sniper cohort, acute low-level blast exposure was associated with reduced global efficiency and focal hypoconnectivity in the pre-supplementary motor area, whereas chronic, career-long exposure showed increased global efficiency and nodal hyperconnectivity in fusiform regions. This acute-to-chronic shift—initial network suppression followed by later hyperconnective reorganization—suggests a potential compensatory adaptation to repeated low-level blast.

Cognitive–motor integration (CMI) paradigms sharpen this picture by taxing concurrent executive control, visuospatial transformation, and motor execution—functions supported by fronto-parietal–cerebellar circuits that are sensitive to diffuse axonal injury and network disconnection [75,134]. In CAF breachers and range staff, CMI testing revealed slower sensorimotor processing and greater intra-individual variability even when standard working-memory indices were unaffected [12,21,75]. These CMI-specific delays have been linked to disrupted coupling between associative and motor cortices and map onto subtle decrements in balance, visuomotor coordination, and dual-tasking—capabilities central to operational readiness [135,136,137]. The selectivity of these effects helps explain why coarse, single-domain tasks (accuracy, simple reaction time) may fail to register change, while integrated, network-demanding tasks do [138]. Together with the latent cognitive changes reported by Di Battista et al. [120] and the context-dependent blast effects observed by Martindale et al. [21], these findings support the view that diffuse network inefficiency often manifests behaviorally only under tasks that tax distributed circuits or when cognitive load is amplified by comorbid conditions—aligning with the present observation that early neural stress is more readily captured by integrated tasks and molecular signatures than by accuracy-based metrics alone.

Mechanistically, preserved performance alongside biomolecular disturbance aligns with established blast pathophysiology [139]. Repetitive low-level blast imposes baromechanical strain on the neurovascular unit, transiently increases BBB permeability, and perturbs glymphatic clearance, initiating chronic neuroinflammatory and oxidative cascades [24,33,47,96,102,140]. In our cohort, elevations in GFAP and PRDX-6 indicate astrocytic activation and redox imbalance; higher tau and VILIP-1 implicate cytoskeletal instability and dysregulated calcium signaling; and modulation of BDNF suggests altered neurotrophic tone and plasticity. These mechanisms plausibly degrade neural efficiency without reducing overt accuracy—particularly in highly trained operators capable of recruiting redundant pathways and cognitive strategies [113,141].

Crucially, compensation is neither cost-free nor indefinite [142]. Network over-recruitment elevates metabolic demand, and chronic reliance on compensatory circuits may accelerate neuroenergetic fatigue—precipitating subtle symptoms (mental tiredness, distractibility, slowed thinking) before standardized tests deteriorate [115]. Electrophysiological studies also show slowed oscillatory synchronization and altered beta-band coherence during cognitive effort, indicating an energetic premium to preserve performance [10,102,143,144]. Longitudinal work in repetitive head-impact and blast cohorts suggests these compensatory states plateau or erode with continued exposure, progressing to processing-speed decline, attentional lapses, and cognitive–motor slowing [21,112,145]. The latent neuroinjury factor identified here likely indexes this subclinical stage—capturing shared variance between biomarkers of astroglial/axonal stress and subjective cognitive-affective symptoms while overt behavior remains stable.

These insights carry methodological and clinical implications. First, reliance on single-domain neuropsychological tests risks false reassurance in high-exposure operators. Multidomain probes that stress integration—CMI tasks, dual-task paradigms, sustained attention under load, and decision-making in complex environments—are more likely to reveal the hidden inefficiency of diffuse network stress [12,21,75,146,147]. Second, coupling such tasks with physiological readouts—task-based fMRI, resting-state connectivity, quantitative EEG/MEG, and oculomotor metrics—can detect compensatory recruitment and altered timing before accuracy declines [5,102,112,130]. Third, integrating blood-based biomarkers adds mechanistic specificity: elevations in GFAP, PRDX-6, VILIP-1, BDNF, and tau isoforms provide convergent evidence of astroglial reactivity, oxidative stress, synaptic dysregulation, and cytoskeletal strain consistent with this compensatory framework [113,147,148,149,150].

Finally, the compensation model offers a path for operational monitoring and intervention. Periodic multidomain assessment—combining biomarker profiling with CMI and network-level neurophysiology—could identify personnel whose performance is maintained by costly compensation, guiding exposure management (e.g., training adjustments, recovery intervals), targeted rehabilitation (aerobic conditioning, vestibular/oculomotor therapy, cognitive pacing), and individualized follow-up. In research contexts, Bayesian latent-state models integrating serial biomarkers with digital cognitive and physiological data can quantify transitions from compensated to decompensated states and estimate dose–response relationships at the individual level [151,152,153].

### 3.5. Integrating Symptom, Neurobiological, and Mechanistic Domains

The latent neuroinjury construct defined in this study unifies biomarker perturbations and symptomatology into a probabilistic expression of subclinical neurobiological stress. Among molecular contributors, BDNF and VILIP-1—key mediators of synaptic plasticity and calcium regulation—showed the strongest loadings, reflecting sensitivity to excitatory imbalance and neuroplastic strain after blast exposure [69,90]. GFAP and PRDX-6, indices of astroglial activation and oxidative stress, likewise featured prominently, underscoring the recurring role of vascular–glial and redox dysregulation in blast pathophysiology [50,96,154]. Together these indicators suggest a coordinated neurobiological response to cumulative baromechanical loading, in which impaired proteostasis, mitochondrial dysfunction, and sustained glial activation contribute to chronic CNS stress.

Biomarker deviations were not uniformly distributed but concentrated in the upper tails, suggesting heterogeneous susceptibility among individuals. Similar “tail-heavy” elevations in p-tau181, NfL, and amyloid-β peptides have been reported in blast-exposed and contact-sport cohorts [73,85,97]. Such skew likely reflects variation in cumulative blast dose, BBB resilience, metabolic capacity, and immune priming [37,38]. Transcriptomic and proteomic studies support this view, showing repeated low-level blast modifies gene networks governing endothelial integrity, Toll-like receptor signaling, oxidative defense, and amyloid-β transport [67,155]. Within this framework, heterogeneity may reflect different positions along a shared cascade of vascular, glial, and proteostatic stress—where some individuals compensate effectively while others shift toward more persistent neuroinflammatory activation.

The symptom dimensions most predictive of the latent factor, difficulties with concentration, irritability, forgetfulness, and slowed thinking, mirror cognitive-affective and fatigue-somatic clusters typical of mTBI/concussion [156]. These experiences may arise from disrupted communication within fronto-limbic and thalamo-cortical networks, regions particularly vulnerable to diffuse axonal injury and glial dysfunction. Functional neuroimaging in blast-exposed personnel demonstrates altered prefrontal, cingulate, and parietal connectivity correlated with symptoms [9,21,130], implicating reduced network efficiency as a plausible bridge between molecular disturbance and subjective experience. Thus, co-expression of biomarker and symptom variance within a single latent dimension reflects a biologically coherent pattern: astroglial and oxidative stress align with network inefficiency manifesting as cognitive fatigue and affective dysregulation, even when task performance remains intact.

Previous CAF breacher studies further support this symptom–biomarker coupling. Astroglial and inflammatory markers (GFAP, MPO, MMP-9) covaried with subjective complaints—particularly cognitive fatigue and irritability—but not with standard neuropsychological scores [45]. Subsequent cohorts found CNS-directed autoantibodies correlated with irritability and slowed thinking, consistent with chronic neuroimmune activation [74]. These associations indicate that subjective symptoms capture a biologically meaningful dimension of CNS strain in populations where compensatory mechanisms may mask overt deficits. Integrating symptom-, biomarker-, and network-level evidence within a unified probabilistic framework therefore offers a cautious but mechanistically grounded approach to detecting early, potentially reversible stages of blast-related neurobiological dysfunction—linking cellular stress to subjective experience before measurable cognitive decline is apparent.

### 3.6. Implications for Clinical Translation and Operational Readiness

Mechanistically, the latent neuroinjury signature reflects a coordinated disturbance of vascular, glial, and metabolic homeostasis that may accrue silently under repetitive blast exposure. The neurobiomarker constellation observed here—elevated GFAP, PRDX-6, tau, VILIP-1, and altered BDNF—broadly aligns with the vascular–glial–tauopathy framework described above and corresponds to histopathological evidence of astroglial scarring, microvascular fragmentation, and perivascular tau deposition in blast-exposed military members and Veterans [2,19,157].

Clinically, this subclinical neurobiological pattern may contribute to the persistent cognitive fatigue, irritability, and affective dysregulation reported in breachers, artillery operators, and SOF personnel. Recognition of this potential prodromal phase highlights an opportunity for earlier characterization and targeted support before overt impairment emerges. Integrating validated biomarker panels—such as GFAP, NfL, PRDX-6, VILIP-1, and emerging markers such as Aβ40/42, p-tau181/217, and brain derived-tau—may enhance sensitivity to underlying neural stress beyond neurocognitive testing or symptom reports alone in high-exposure populations [103,158]. Importantly, such approaches are most feasible within specialized or high-risk units, pilot programs, or targeted surveillance initiatives, rather than force-wide implementation.

Operationally, these findings highlight the value of multimodal, precision-health frameworks that pair blood-based biomarkers with advanced neuroimaging, digital cognitive tools, and wearable blast dosimetry [67,155]. Such integrated systems may help refine recovery intervals, inform exposure limits, and support objective return-to-duty decisions while enabling targeted interventions to sustain performance. Alignment with NATO and Five-Eyes initiatives focused on biomarker harmonization and exposure standardization would further strengthen the translational utility of this approach [65,159].

Ultimately, embedding biomarker-based assessments within routine occupational health monitoring may help bridge mechanistic neuroscience and operational medicine, enabling early characterization of neurobiological strain and supporting evidence-informed strategies to mitigate long-term neurological risk and sustain operational readiness [53,66].

### 3.7. Limitations and Future Directions

Several limitations should be considered when interpreting these findings. First, the cross-sectional design and modest, occupation-specific sample preclude causal inference and limit generalizability beyond similarly exposed military populations and is susceptible to erroneous causal assumptions. Although blast exposure was well characterized at the occupational level for this cohort, individual-level blast overpressure was not directly quantified. The absence of wearable blast telemetry—reflecting practical and logistical constraints during training—limits precise dose–response modeling and constrains mechanistic interpretation. Future acute exposures and longitudinal studies integrating validated personal dosimetry with repeated biomarker and performance assessments will be essential to resolve exposure thresholds, recovery trajectories, and inter-individual susceptibility.

Second, while the present analysis focused on working memory as an index of performance resilience, this domain alone may underestimate subtle or context-dependent impairments. Prior work suggests that executive control, sustained attention, cognitive–motor integration, and decision-making under load may be more sensitive to diffuse network inefficiency associated with chronic low-level blast exposure. Expanding neurobehavioral assessment batteries, particularly under conditions of increased cognitive or physiological load, will be important for capturing compensatory limits that may not be evident in single-domain or low-demand tasks.

Third, self-reported symptoms contributed more strongly to the latent neuroinjury construct than either cognitive performance or individual biomarkers. This pattern underscores the relevance of subjective symptom burden in subclinical, high-functioning populations, while also warranting caution in interpreting latent constructs that integrate heterogeneous data streams. Multimodal frameworks that combine circulating biomarkers with neuroimaging, digital performance analytics, and physiological measures may better resolve how neural efficiency, compensatory mechanisms, and symptom expression interact across different stages of exposure and recovery. For example, incorporation of continuous physiological monitoring, such as wearable-derived exertion, recovery, and autonomic metrics, may improve sensitivity to compensatory strain and enable more precise optimization of exposure–recovery balance in operational settings.

Finally, although the biomarker-informed framework presented here shows promise for characterizing neurobiological strain in high-exposure personnel, its scalability and operational feasibility will depend on careful prioritization of markers, cost–benefit considerations, and integration with existing health surveillance systems. Future efforts should emphasize parsimonious, harmonized monitoring strategies aligned with allied initiatives, enabling translation from research cohorts to broader operational force health protection.

## 4. Materials and Methods

### 4.1. Participants

This prospective, cross-sectional cohort study enrolled Canadian Forces School of Military Engineering (CFSME) breacher instructors and range staff (*n* = 18; hereafter referred to as *Breachers*) with extensive occupational exposure to repetitive low-level blast overpressure accrued across their military careers. These personnel routinely conduct and supervise controlled explosive breaching activities as part of training and instructional duties.

The control group comprised CAF personnel (*n* = 19) with comparable age and sex distributions and no occupational history of blast exposure. Controls were selected to be similar to Breachers with respect to military experience, work schedules, and exposure to general operational, physical, and psychological stressors, differing primarily in the absence of routine blast exposure, as reported previously [12].

No participants in either group reported a clinically diagnosed history of moderate or severe TBI, major neurological disorder, psychotic illness, learning disability, or current substance abuse. Lifetime history of mild TBI/concussion was assessed by self-report in both groups. A subset of participants in each group reported remote, self-limited concussions earlier in their careers, as previously described [12,45]; the prevalence and distribution of head injury history were comparable between Breachers and Controls and were not associated with current symptom burden. Participants were excluded if they were taking medications known to affect cognitive or neurological function, or if they were acutely symptomatic due to infection, illness, or seasonal allergies at the time of testing.

The study was approved by the Defence Research and Development Canada (DRDC) Human Research Ethics Committee (HREC Protocol No. 2016-006) and conducted in accordance with the ethical principles of the Declaration of Helsinki. All participants received a detailed explanation of study procedures and potential risks and provided written informed consent prior to participation. Participant demographics and selected military characteristics are summarized in Table 1.

### 4.2. Experimental Design and Procedures

Data collection for CFSME Breachers and Controls was conducted during a single visit at one of two locations: Canadian Forces Base Gagetown (CFB Gagetown, NB) for Breachers and Defence Research and Development Canada–Toronto Research Centre (DRDC-TRC, ON) for controls. The identical protocol was followed at both sites. Following informed consent, participants provided demographic information and a comprehensive health and medical history, including details on operations (e.g., number of war-zone deployments, exposure to blasts, and proximity to explosions) and any history of concussions or brain injuries. Participants also completed occupational questionnaires about breaching and explosive experience, including intensities and frequencies of exposure.

Although individual-level blast overpressure telemetry was not collected in the present cohort, exposure characterization was informed by occupational role and prior empirical assessments conducted within the same training environment. Breacher instructors and range staff at the CFSME routinely supervise and participate in repeated controlled detonations during breaching training exercises. Importantly, DRDC-led field measurements using blast gauges (BlackBox Biometrics Inc., Rochester NY, USA), with sensors positioned on the helmet, non-firing shoulder, and chest, were previously conducted in a comparable cohort of CFSME instructors and range staff performing similar activities on the same breaching range. These assessments demonstrated that more than 30% of recorded blast wave events exceeded the commonly cited 4 psi (27.6 kPa) threshold during routine training operations [14,160]. Although these measurements were not acquired contemporaneously with the present study and do not permit direct estimation of individual cumulative blast dose, they provide relevant contextual evidence that CFSME breacher instructors are repeatedly exposed to BOP within a range previously associated with adverse alterations in brain health and performance in both human and experimental studies [161,162]. Accordingly, the occupational role–based exposure classification used here reflects a real-world training environment characterized by documented, repetitive exposure to biologically meaningful blast intensities.

Consistent with this exposure context, participants in the current breacher group typically contributed to 8–20 breaching training courses per year, each comprising 1–2 days on the breaching range with more than six controlled detonations per day. Breachers commonly delivered these courses over periods of one to three years, resulting in substantial cumulative exposure of variable frequency and magnitude [12]. To minimize potential confounding from acute physiological effects, participants were instructed to abstain from blast exposure for at least 48 h and to avoid strenuous physical activity for 24 h prior to testing. All data were de-identified and securely stored in a protected database to ensure participant confidentiality and data integrity. None of the participants reported a history of acquired brain injury, major neurological or psychiatric disorder, learning disability, or active substance abuse.

#### 4.2.1. Rivermead Post-Concussion Symptoms Questionnaire

We administered the Rivermead Post Concussion Symptoms Questionnaire to measure post-concussive symptoms [163,164]. The RPQ includes 16 items, each of which represents a symptom associated with concussion. For each symptom, participants were asked to indicate whether they had experienced it as a function of injury to the head using a 5-point scale (0 = not experienced at all, 4 = a severe problem).

#### 4.2.2. N-Back Test of Working Memory

This task assesses working-memory updating and maintenance across three load levels (1–3 back) [165]. Performance sensitivity was quantified using *d′* derived from signal detection theory [166], where higher values denote greater discriminability and *d′* ≈ 0 reflects chance-level performance. Because *d′* is undefined when hit rates are 100% or false-alarm rates are 0% (i.e., when probability terms cannot be computed), standard correction procedures were applied. Participants with perfect hit rates or zero false alarms were reassigned adjusted values of 0.99 and 0.01, respectively, yielding a maximum possible *d′* of 4.65. These corrections ensure stable estimation of sensitivity across participants and load conditions.

### 4.3. Blood Sample Collection, Processing and Storage

Peripheral blood samples were collected from participants in a fasting state by a trained medical technologist using standard phlebotomy procedures. Venous blood was drawn into 10 mL K_2_EDTA tubes (BD Vacutainer^®^, Franklin Lakes, NJ, USA), promptly centrifuged at 1600× *g* for 15 min at 4 °C, and separated into plasma aliquots for storage at −80 °C until further analysis. To maintain consistency, all samples were processed uniformly at the same time of day.

### 4.4. Plasma Neurological Biomarker Analyses

A panel of 13 neurological injury biomarkers was selected based on their brain specificity and potential to reflect distinct injury mechanisms: (1) neuronal cell body damage [neuron-specific enolase (NSE), neurogranin (NRGN), visinin-like protein (VILIP)-1, ubiquitin carboxy-terminal hydrolase (UCH-L1)]; (2) diffuse axonal injury [neurofilament light chain (NF-L), phosphorylated neurofilament heavy chain (pNF-H)]; (3) astroglial damage/gliosis [S100 calcium-binding protein beta (S100B), glial fibrillary acidic protein (GFAP)]; (4) cerebral ischemia/oxidative stress [creatine kinase brain isoenzyme (CK-BB), peroxiredoxin-6 (PRDX-6)]; (5) neuroinflammation [monocyte chemoattractant protein (MCP-1)]; (6) neural repair/plasticity [brain-derived neurotrophic factor (BDNF)]; (7) chronic neurodegeneration [total tau (T-tau]). Biomarkers were quantified using a combination of ultra-sensitive multiplexed neurological immunoassay panels from MesoScale Diagnostics, LLC (MSD^®^, Gaithersburg, MD, USA) MULTI-ARRAY™ Human Electrochemiluminescence assay and SINGLE MOLECULE ARRAY (SiMoA™) Human Neurology 4-Plex assay (Quanterix^®^, Lexington, MA, USA), as previously reported [101,167,168,169,170,171]. Biomarker values were considered usable if they were within the detection limits specified by the manufacturer and displayed a coefficient of variation (CV) of <20% between duplicate samples. All biomarkers were analyzed neat and in duplicate, with the mean value of the duplicates used as the final value. To minimize assay variation, all specimens were analyzed on the same day, in random order, by the same technician who was blinded to the participant’s status. Limits of detection (LLOD) and dynamic ranges are available on the MSD website (https://www.mesoscale.com/en (accessed 20 November 2025)).

### 4.5. Data Analysis

#### 4.5.1. Workflow

**Figure 6:**
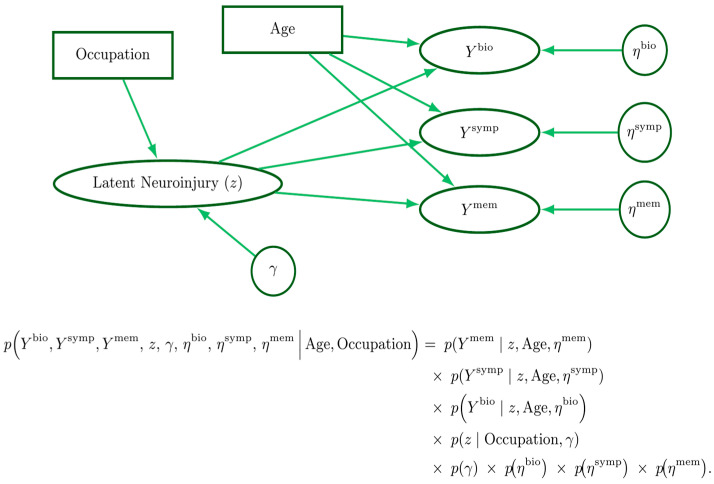
A narratively generative variate–covariate model. Rectangles represent fixed covariates, ellipses denote variates (outcomes), and circles represent parameters. The latent neuroinjury () node is drawn as an ellipse for convenience, as it is both a parameter and variate. Beneath the graphical model is the corresponding joint probability factorization.

The data analysis followed principled Bayesian workflow practices outlined by McElreath [108] and Betancourt [107]. The workflow began with a graphical model (Figure 6) that made explicit all scientific assumptions derived from domain expertise, providing a transparent foundation for subsequent statistical decisions. From this foundation, we constructed a prior-only statistical model that encoded our beliefs about plausible parameter values and outcome ranges. Prior predictive simulations were then used to calibrate the model, allowing us to detect pathologies, refine priors, and validate that the generative assumptions could reproduce biologically and clinically credible patterns. We then fit the model to simulated data to evaluate parameter recovery and check for redundancies and degeneracies. After iterating between these stages as needed, we fit the model to observed data and evaluated its adequacy with posterior retrodictive checks.

#### 4.5.2. Scientific Modeling Assumptions

The primary aim of this study was two-fold: (1) to characterize a latent neuroinjury process that integrates molecular markers of brain injury, chronic brain injury symptoms, and measures of working memory, and (2) to quantitatively compare this process between military members in occupations with presumed higher repetitive exposure to low-level blast (breachers) and their non-exposed colleagues. Our scientific model followed the assumption that ‘neuroinjury’, could be quantified as a single metric informed by blood levels of biomarkers commonly associated with neuroinjury, self-reported post-concussion/brain injury symptoms, and the N-Back test of working memory. We hypothesized that breachers, due to their history of blast exposure, would display elevated levels of this latent neuroinjury factor compared to non-breacher counterparts. In, addition, we considered that age could influence each of the neuroinjury domains (biomarkers, symptoms, memory), independently of occupational exposure.

#### 4.5.3. Statistical Modeling Assumptions

Our statistical model was constructed to reflect the above scientific assumptions. In addition, we assumed that the biomarker and symptom domains could both be modeled as ordinal outcomes arising from underlying continuous distributions. Specifically, biomarkers were divided into roughly equal tertiles corresponding to low, medium, and high categories. This approach served two purposes. First, it addressed the presence of values below the limit of assay detection. Second, it acknowledged our assumption that small numeric differences in biomarker levels are unlikely to have profound implications, whereas broader categorical distinctions may better reflect biologically and clinically relevant variation. This is an especially relevant assumption in small sample sizes like the current study, where ordinal categorization produces a sensible trade off of precision for stability, limiting the influence of biological noise and extreme values.

Symptom responses on the RPQ were collapsed from five categories to three: Low (0/1), Medium (1/2), Severe (3/4). This decision was made to mitigate sparse categories, stabilize threshold estimation in a small sample, and improve interpretability.

Finally, we modeled performance on the 1-, 2-, and 3-back tasks jointly using a multivariate normal likelihood to account for within-subject correlations across tests. This was motivated by the fact that N-back performance was summarized using signal-detection sensitivity (*d′*), defined as the difference between z-transformed hit rates and false alarm rates, which yields continuous scores.

A visual diagram of the combined scientific and statistical modeling assumptions in variate-covariate from [172], as well as the full joint conditional model can be seen in Figure 6.

#### 4.5.4. Data

The dataset for this study consisted of a 37 row by 39 column matrix including seven participant demographic and characteristic variables (Group, Sex, Age, Military Status, Rank, years Breaching, Warzone Deployment), three N-Back variables (-1, -2, -3 Back *d′*), 13 Biomarkers, and 16 RPQ questions.

#### 4.5.5. Estimands (Quantities of Interest)

Our primary estimands were (1) the individual-level latent neuroinjury score defined jointly by biomarkers, symptoms, and memory performance; and (2) the group-level contrast in these scores between Breachers and Controls. A secondary estimand was the individual loadings or contributions of each item within each domain (biomarkers, symptoms, memory) to the latent neuroinjury score.

#### 4.5.6. Estimators (Statistical Models Used)

A linear regression was used to model individual-level latent neuroinjury scores (Equation (1)), with group membership as the sole covariate. Here, $\mu_{z,i}$ is the latent neuroinjury score for individual i, modeled with unit variance for scale identification. $\mu_{z,i}$ is the expected latent score, determined by an intercept α and the group indicator $G_{i}$ $\left(G_{i}=1\right)$ for breachers, $\left(G_{i}=0\right)$ for non breachers. $\beta_{\mathrm{occupation}}$ represents the group effect, interpreted in standard deviation units of the latent scale. Weakly informative priors were placed on the α and β parameters:

(1) $$\begin{array}{c} z_{i}∼N\left(\mu_{z,i},\;1\right)\\ \mu_{z,i}=\alpha +\beta_{\mathrm{occupation}}\;G_{i}\\ \alpha ∼N\left(0,\;0.3/2.32\right)\\ \beta_{\mathrm{occupation}}∼N\left(0,\;1/2.32\right) \end{array}$$

Biomarkers (Equation (2)) and RPQ symptoms (Equation (3)) were treated as ordered categorical outcomes and modeled using ordinal logistic regression, with age and the latent neuroinjury scores as covariates. This specification followed an item-response theory framework: each biomarker or symptom was an “item” whose probability of occupying a higher category depends on the individual’s latent neuroinjury score and age, with item-specific coefficients indicating how strongly each item reflects variation in the latent neuroinjury process. Individuals were indexed by i=1,…,N. Biomarker “items” were indexed by b=1,…,B and RPQ symptom items by q=1,…,Q. Age for individual i is $A_{i}$ and $A_{0}$ is the reference age (e.g., the sample mean) used for mean-centering. The parameter $eta^{i}$ represents the linear model inside the cumulative link function for each biomarker or RPQ item, with c representing the cutpoints. Parameters $\beta_{Z}$ and $\beta_{A}$ represent the latent neuroinjury and age covariates, respectively.

(2) $$\begin{array}{c} Y_{ib}^{\mathrm{bio}}∼OrderedLogit\left(\eta_{ib}^{\mathrm{bio}},\;c_{b}^{\mathrm{bio}}\right)\\ \eta_{ib}^{\mathrm{bio}}=\beta_{b,Z}^{\mathrm{bio}}\;z_{i}+\beta_{b,A}^{\mathrm{bio}}\;\left(A_{i}-A_{0}\right)\\ \beta_{b,Z}^{\mathrm{bio}}∼N\left(0,\;1/2.57\right)\\ \beta_{b,A}^{\mathrm{bio}}∼N\left(0,\;1/2.32\right)\\ p_{b}^{\mathrm{bio}}∼Dirichlet\left(2\right)\\ c_{bk}^{\mathrm{bio}}=logit\left(\sum_{j=1}^{k}p_{bj}^{\mathrm{bio}}\right),\;k=1,\ldots ,K_{\mathrm{bio}}-1 \end{array}$$

(3) $$\begin{array}{c} Y_{iq}^{\mathrm{rpq}}∼OrderedLogit\left(\eta_{iq}^{\mathrm{rpq}},\;c_{q}^{\mathrm{rpq}}\right)\\ \eta_{iq}^{\mathrm{rpq}}=\beta_{q,Z}^{\mathrm{rpq}}\;z_{i}+\beta_{q,A}^{\mathrm{rpq}}\left(A_{i}-A_{0}\right)\\ \beta_{q,Z}^{\mathrm{rpq}}∼N\left(0,\;1/2.57\right)\\ \beta_{q,A}^{\mathrm{rpq}}∼N\left(0,\;1/2.32\right)\\ p_{q}^{\mathrm{rpq}}∼Dirichlet\left(2\right)\\ c_{qk}^{\mathrm{rpq}}=logit\left(\sum_{j=1}^{k}p_{qj}^{\mathrm{rpq}}\right),\;k=1,\ldots ,K_{\mathrm{rpq}}-1 \end{array}$$

N-Back performance $Y^{\mathrm{nback}}it$ for tasks t=1,2,3 was modeled jointly with a multivariate normal likelihood to account for within-subject correlation. Where $\alpha_{t}^{\mathrm{nback}}$ are task-specific intercepts, $\beta_{t,Z}^{\mathrm{nback}}\;z_{i}$ are loadings on the latent neuroinjury score $z_{i}$, and $\beta_{t,A}^{\mathrm{nback}}\left(A_{i}-A_{0}\right)$ are mean centered age effects. The covariance across tasks was parameterized as $\Sigma =diag\left(\sigma \right)Rdiag\left(\sigma \right)$, with task-specific standard deviations $\sigma_{t}$ and correlation matrix R assigned an LKJ prior.

(4) $$\left(\begin{array}{c} Y_{i,1}^{1\mathrm{back}}\\ Y_{i,2}^{2\mathrm{back}}\\ Y_{i,3}^{3\mathrm{back}} \end{array}\right)∼MVNormal\left(\left(\begin{array}{c} \mu_{i,1}^{1\mathrm{back}}\\ \mu_{i,2}^{2\mathrm{back}}\\ \mu_{i,3}^{3\mathrm{back}} \end{array}\right),\;\Sigma \right)\;\mu_{i,t}^{\mathrm{nback}}=\alpha_{t}^{\mathrm{nback}}+\beta_{t,Z}^{\mathrm{nback}}\;z_{i}+\beta_{t,A}^{\mathrm{nback}}\left(A_{i}-A_{0}\right),\;t=1,2,3\;\alpha^{1\mathrm{back}}∼N\left(4,\;1/2.32\right),\;\alpha^{2\mathrm{back}}∼N\left(3,\;1/2.32\right),\;\alpha^{3\mathrm{back}}∼N\left(1.5,\;1/2.32\right)\;\beta_{t,Z}^{\mathrm{nback}}∼N\left(0,\;1/2.57\right),\;\beta_{t,A}^{\mathrm{nback}}∼N\left(0,\;1/2.32\right),\;\sigma_{t}∼Exponential\left(1\right),\;R∼LKJ\left(2\right),\;\Sigma =diag\left(\sigma \right)\;R\;diag\left(\sigma \right)$$

#### 4.5.7. Estimates (Posterior Inferences)

Posterior samples were generated via Hamiltonian Monte Carlo (HMC) as implemented in STAN [173]. These samples approximate the posterior distribution, and reported results are summaries of these draws. Typically, estimates are presented as posterior means with 90% credible intervals (CrIs). The summaries correspond to parameters or derived quantities constructed from the posterior parameter draws. For the latent neuroinjury score, group expectations were summarized directly from the derived μ of the linear model in Equation (1), and the group contrast was obtained by subtracting expectations between groups at each draw. Latent neuroinjury item loadings were summarized as the mean and 90% CrIs of the latent neuroinjury parameters for each item. For biomarkers and RPQ items, expected group differences were obtained by marginalizing the posterior predictive distribution of category probabilities, conditional on group differences in the latent neuroinjury score. At each posterior draw, predicted probabilities of falling in each category were computed, and group contrasts were summarized by their posterior means, 90% CrIs, and posterior probability that the contrast was greater than zero. For N-Back tasks, posterior draws of the regression parameters were used to generate predicted group-specific expectations (μ) for each task from the multivariate normal model. Group contrasts were then calculated and summarized analogously with posterior means, 90% CrIs, and the posterior probability that the contrast was greater than zero.

#### 4.5.8. Software

The software Stan (Version 0.8.0) [173] provided the HMC engine which was interfaced through CmdStanR (run on R (Version 4.5.1) [174], using the RStudio integrated development environment (Version 2025.9.0.387) [174]. Model checks were aided by the model checking and plotting utilities created by Michael Betancourt [175]. To aid in the summarizing and visualizing of posterior samples the R package ‘rethinking’ was used [176]. Tables were made using the ‘gt’ [177] and ‘gtsummary’ [178] packages, with aid from the ‘dplyr’ [179] ‘tidyr’ [180] and ‘stringr’ [181] packages. The neuroinjury loadings plot was created using ‘ggplot2’ [182] with the aid of the ‘cowplot’, [183] and ‘forcats’ [184] packages (all accessed 20 November 2025). The ‘TinyTex’ [185] ‘magick’ [186] and ‘pdftools’ [187] packages were used to create the variate-covariate diagram seen in Figure 6 (all accessed 20 November 2025).

#### 4.5.9. Reproducibility

Code and augmented data used for all statistical modeling in this manuscript can be found in a public GitHub repository (https://github.com/dibatti5/dibattista_et_al_2025_latent_neuroinjury_in_military_breachers, accessed on 22 November 2025).

## 5. Conclusions

This study provides probabilistic evidence that military breachers with extensive occupational exposure to low-level blast overpressure may exhibit a latent neurobiological profile characterized by modest but coordinated elevations in symptom burden and select circulating neuroinjury biomarkers, occurring in the context of preserved working-memory performance. The multidomain pattern observed—integrating post-concussive symptoms with biomarkers related to astroglial activity, oxidative stress, and cytoskeletal integrity—is broadly consistent with prior human and experimental research describing vascular–glial contributions to cumulative blast-related neural strain, while remaining subclinical and heterogeneous in expression. Importantly, cognitive performance measures did not contribute substantively to the latent construct, reinforcing a dissociation between molecular and symptomatic signals and overt task impairment.

By applying a Bayesian latent-variable framework, this work demonstrates a principled and transparent approach for integrating symptom, cognitive, and biomarker data into a unified probabilistic construct, enabling characterization of subtle neurobiological strain that may not be captured by single-domain analyses alone. While preliminary and not intended for diagnostic application, this framework provides a scalable foundation for future longitudinal investigations incorporating objective exposure metrics and repeated assessments. Taken together, these findings suggest that probabilistic integration of molecular indicators and symptom profiles may have translational relevance for occupational health surveillance, individualized risk stratification, and longitudinal monitoring in military personnel routinely exposed to repetitive low-level blast, while underscoring the need for continued validation in larger, dose-informed cohorts.

## Figures and Tables

**Table 1:** Participant demographics and service characteristics.

Characteristic	Breacher (*N* = 18)	Control (*N* = 19)
Sex		
Female	2 (11%)	2 (11%)
Male	16 (89%)	17 (89%)
Age	32.0 (26.0, 37.0)	32.0 (27.0, 36.0)
Status		
Primary Reserve	9 (50%)	11 (58%)
Regular Force	9 (50%)	8 (42%)
Rank		
Junior NCM	5 (28%)	13 (68%)
Senior	11 (61%)	0 (0%)
Subordinate Officer	0 (0%)	0 (0%)
Junior Officer	2 (11%)	6 (32%)
Senior Officer	0 (0%)	0 (0%)
General Officer	0 (0%)	0 (0%)
Years of Breaching	6.5 (4.0, 10.0)	0.0 (0.0, 0.0)
War Zone Deployment	10 (63%)	0 (0%)
N-Back Prime		
1-Back	4.7 (4.0, 4.7)	4.0 (3.4, 4.7)
2-Back	2.6 (2.2, 3.1)	2.8 (2.1, 3.6)
3-Back	1.2 (0.8, 1.7)	1.3 (1.2, 1.8)

## Data Availability

Aggregate, group-level data supporting the findings of this study are included within the article and its Supplementary Materials. In addition, a GitHub repository containing the analysis code and non-identifiable supporting materials is available. Individual-level datasets generated and analyzed during the study are not publicly available due to privacy, ethical, and governance restrictions associated with research involving Department of National Defence (DND) and Canadian Armed Forces (CAF) personnel. Specifically, variables such as study site, dates of data collection, date of birth, and participant gender could permit re-identification. De-identified datasets with these variables removed may be made available by the corresponding author upon reasonable request, subject to institutional ethics approval and applicable data protection policies.

## Supplementary Materials

The following supporting information can be downloaded at: https://www.mdpi.com/article/10.3390/ijms27020592/s1.

## Abbreviations

Aβ	Amyloid-β
Aβ40/42	Amyloid-β 40 and 42 peptides
AD	Alzheimer’s disease
ANAM	Automated Neuropsychological Assessment Metrics
APP	Amyloid precursor protein
ATP	Adenosine triphosphate
BBB	Blood–brain barrier
BDNF	Brain-derived neurotrophic factor
BD-tau	Brain-derived tau
BOP	Blast overpressure
CAF	Canadian Armed Forces
CI	Credible interval
CMI	Cognitive–motor integration
CNS	Central nervous system
CREB	cAMP response element-binding protein
CSF	Cerebrospinal fluid
CTE	Chronic traumatic encephalopathy
DTI	Diffusion tensor imaging
EEG	Electroencephalography
EV	Extracellular vesicle
fMRI	Functional magnetic resonance imaging
GFAP	Glial fibrillary acidic protein
IL-6	Interleukin-6
IRR	Injury risk ratio (if present)
JNK	c-Jun N-terminal kinase
MAPK	Mitogen-activated protein kinase
MBP	Myelin basic protein
MCP-1	Monocyte chemoattractant protein-1
MEG	Magnetoencephalography
MMP	Matrix metalloproteinase
mBDNF	Mature BDNF
mTBI	Mild traumatic brain injury
NfL	Neurofilament light chain
pNF-H	Phosphorylated neurofilament heavy chain
NGRN	Neurogranin
NSE	Neuron-specific enolase
PET	Positron emission tomography
PCL	PTSD Checklist
PI3K	Phosphoinositide 3-kinase
PLCγ	Phospholipase C-gamma
PRDX-6	Peroxiredoxin-6
PTSD	Post-traumatic stress disorder
RBANS	Repeatable Battery for the Assessment of Neuropsychological Status
RPQ	Rivermead Post-Concussion Questionnaire
S100b	S100 calcium-binding protein beta
SOF	Special Operations Forces
T-tau	Total tau
TBI	Traumatic brain injury
TNF-α	Tumor necrosis factor-alpha
TrkB	Tropomyosin receptor kinase B
UCH-L1	Ubiquitin carboxy-terminal hydrolase L1
VCAM-1/ICAM-1	Vascular/Intercellular cell adhesion molecules
VILIP-1	Visinin-like protein 1
