# Supplementary material for: Integrated Blood Biomarker and Neurobehavioural Signatures of Latent Neuroinjury in Experienced Military Breachers Exposed to Repetitive Low-Intensity Blast
Source: Int J Mol Sci. 2026 Jan 6;27(2):592. doi: 10.3390/ijms27020592 (PMC12840665; doi:10.3390/ijms27020592)
Supplement: Supplementary file 1 [file ijms-27-00592-s001.zip › Table S2-biomarker_raw_means.pdf]

Table S2. Biomarker Levels

Characteristic	Breacher N = 18[1]	Non-Breacher N = 19[1]
**BDNF**	358.6 (181.6, 586.8)	181.4 (103.7, 298.6)
**CKBB**	1,113.7 (732.2, 1,396.5)	909.9 (588.7, 1,007.8)
**GFAP**	100.4 (81.0, 121.1)	61.0 (52.4, 81.0)
**NRGN**	500.7 (133.1, 918.6)	450.5 (362.0, 615.8)
**NSE**	932.9 (673.7, 1,132.9)	843.9 (746.1, 1,006.6)
**PRDX6**	20,015.4 (14,754.2, 28,614.6)	13,260.7 (10,880.4, 16,382.1)
**s100B**	1,005.7 (851.1, 1,231.3)	744.5 (688.9, 806.3)
**Tau**	20.8 (16.5, 25.6)	13.6 (11.0, 15.1)
**VILIP-1**	27.9 (19.3, 44.0)	18.3 (14.9, 30.2)
**MCP-1**	118.4 (101.6, 132.2)	97.6 (76.7, 109.0)
**UCH-L1**	19.7 (11.0, 32.9)	6.9 (4.5, 8.5)
**NF-L**	3.7 (3.1, 5.9)	3.7 (3.1, 4.3)
**pNF-H**	81.7 (63.0, 96.8)	61.9 (51.5, 79.5)

[1] Median (Q1, Q3)
