# Supplementary material for: Integrated Blood Biomarker and Neurobehavioural Signatures of Latent Neuroinjury in Experienced Military Breachers Exposed to Repetitive Low-Intensity Blast
Source: Int J Mol Sci. 2026 Jan 6;27(2):592. doi: 10.3390/ijms27020592 (PMC12840665; doi:10.3390/ijms27020592)
Supplement: Supplementary file 1 [file ijms-27-00592-s001.zip › Table S1-biomarker groups.pdf]

Table S1. Biomarker Groups

Biomarker / Category (pg/mL)	Group	
	Breacher[1]	Non-Breacher[1]
**BDNF**		
Low (≤ 181.30 pg/mL)	4 (22%)	9 (47%)
Med (>181.30–≤ 328.50 pg/mL)	5 (28%)	7 (37%)
High (>328.50 pg/mL)	9 (50%)	3 (16%)
**CKBB**		
Low (≤ 746.40 pg/mL)	5 (28%)	8 (42%)
Med (>746.40–≤ 1,049.70 pg/mL)	4 (22%)	8 (42%)
High (>1,049.70 pg/mL)	9 (50%)	3 (16%)
**GFAP**		
Low (≤ 62.10 pg/mL)	2 (11%)	11 (58%)
Med (>62.10–≤ 97.50 pg/mL)	5 (28%)	7 (37%)
High (>97.50 pg/mL)	11 (61%)	1 (5.3%)
**NRGN**		
Low (≤ 377.10 pg/mL)	7 (39%)	6 (32%)
Med (>377.10–≤ 585.60 pg/mL)	5 (28%)	7 (37%)
High (>585.60 pg/mL)	6 (33%)	6 (32%)
**NSE**		
Low (≤ 748.30 pg/mL)	7 (39%)	6 (32%)
Med (>748.30–≤ 1,006.60 pg/mL)	3 (17%)	9 (47%)
High (>1,006.60 pg/mL)	8 (44%)	4 (21%)
**PRDX6**		
Low (≤ 12,617.90 pg/mL)	4 (22%)	9 (47%)
Med (>12,617.90–≤ 18,531.20 pg/mL)	3 (17%)	9 (47%)
High (>18,531.20 pg/mL)	11 (61%)	1 (5.3%)
**s100B**		
Low (≤ 744.50 pg/mL)	3 (17%)	10 (53%)
Med (>744.50–≤ 986.70 pg/mL)	5 (28%)	7 (37%)
High (>986.70 pg/mL)	10 (56%)	2 (11%)
**Tau**		
Low (≤ 13.90 pg/mL)	1 (5.6%)	12 (63%)
Med (>13.90–≤ 17.10 pg/mL)	6 (33%)	6 (32%)
High (>17.10 pg/mL)	11 (61%)	1 (5.3%)
**VILIP-1**		
Low (≤ 18.34 pg/mL)	3 (17%)	10 (53%)

Biomarker / Category (pg/mL)	Group	
	Breacher[1]	Non-Breacher[1]
Med (>18.34–≤ 29.52 pg/mL)	8 (44%)	4 (21%)
High (>29.52 pg/mL)	7 (39%)	5 (26%)
**MCP-1**		
Low (≤ 97.60 pg/mL)	3 (17%)	10 (53%)
Med (>97.60–≤ 116.70 pg/mL)	6 (33%)	6 (32%)
High (>116.70 pg/mL)	9 (50%)	3 (16%)
**UCH-L1**		
Low (≤ 7.14 pg/mL)	4 (22%)	13 (68%)
Med (>7.14–≤ 14.50 pg/mL)	4 (22%)	6 (32%)
High (>14.50 pg/mL)	10 (56%)	0 (0%)
**NF-L**		
Low (≤ 3.28 pg/mL)	12 (67%)	11 (58%)
Med (>3.28–≤ 4.14 pg/mL)	2 (11%)	5 (26%)
High (>4.14 pg/mL)	4 (22%)	3 (16%)
**pNF-H**		
Low (≤ 61.41 pg/mL)	4 (22%)	9 (47%)
Med (>61.41–≤ 83.81 pg/mL)	6 (33%)	6 (32%)
High (>83.81 pg/mL)	8 (44%)	4 (21%)

[1] n (%)
